# Implementation of a Capillary Blood Self-Sampling Technique at Home for Monitoring of Patients With IBD

**DOI:** 10.1093/ibd/izaf240

**Published:** 2025-10-31

**Authors:** Gillian S Schuurman, Wouter Tiel Groenestege, Meike M C Hirdes, Herma H Fidder, Bas Oldenburg, Sytze de Roock, Fiona D M van Schaik

**Affiliations:** Department of Gastroenterology and Hepatology, University Medical Center Utrecht, Utrecht, the Netherlands; Utrecht University, Utrecht, the Netherlands; Utrecht University, Utrecht, the Netherlands; Central Diagnostic Laboratory, University Medical Center Utrecht, Utrecht, the Netherlands; Department of Gastroenterology and Hepatology, University Medical Center Utrecht, Utrecht, the Netherlands; Utrecht University, Utrecht, the Netherlands; Department of Gastroenterology and Hepatology, University Medical Center Utrecht, Utrecht, the Netherlands; Utrecht University, Utrecht, the Netherlands; Department of Gastroenterology and Hepatology, University Medical Center Utrecht, Utrecht, the Netherlands; Utrecht University, Utrecht, the Netherlands; Utrecht University, Utrecht, the Netherlands; Department of Pediatric Immunology and Rheumatology, Division of Pediatrics, University Medical Center Utrecht, Utrecht, the Netherlands; Department of Gastroenterology and Hepatology, University Medical Center Utrecht, Utrecht, the Netherlands; Utrecht University, Utrecht, the Netherlands

**Keywords:** remote care, disease monitoring, laboratory diagnostics, inflammatory bowel disease

## Abstract

**Background:**

Remote healthcare aims to improve the management of inflammatory bowel disease (IBD) patients by reducing hospital visits. This is the first study to assess capillary blood sampling at home for routine measurement of chemistry parameters and complete blood count parameters at several time points for disease monitoring in IBD patients.

**Methods:**

In this prospective, single-center proof-of-concept study, 27 patients with Crohn’s disease or ulcerative colitis and an indication for frequent blood monitoring performed capillary blood sampling in the hospital (time point 0 [T0]) and at 2 time points at home (T1 and T2). A successful at home sampling was defined as a blood sample that was (1) transported in <48 hours, (2) of sufficient quality, and (3) a sufficient volume.

**Results:**

A total of 21 patients completed the study (mean age 31 years; 69% Crohn’s disease, 31% ulcerative colitis). Seventeen (81%) out of 21 and 20 (95%) out of 21 blood samples were successfully analyzed, at T1 (between 2 and 6 weeks after T0) and T2 (between 6 and 12 weeks after T0), respectively. At T2, 12 (57%) out of 21 patients preferred capillary blood sampling at home over venous sampling at the hospital. Younger patients expressed higher satisfaction rates. Fifteen (71%) out of 21 patients reported a better performance with blood sampling at T2 compared with T1.

**Conclusions:**

This study shows a high success rate for capillary blood sampling at home for routine disease monitoring in IBD patients. Device optimization and identification of patient preferences are needed to effectively integrate blood sampling at home in remote monitoring of IBD patients.

Key Messages *What is already known?*

- Inflammatory bowel disease (IBD) monitoring through remote symptom monitoring with eHealth technologies and calprotectin testing has improved IBD care, but routine blood monitoring still necessitates laboratory visits.

*What is new here?*

- The results of this study show high success rates for capillary blood sampling at home for routine IBD blood tests, at several time points, as required for daily practice.

*How can this study help patient care?*

- When added to eHealth technologies, blood sampling at home has the potential to enable full remote monitoring of IBD patients, thereby reducing inconvenience for patients, work productivity loss, and healthcare utilization.

## Introduction

Inflammatory bowel disease (IBD) is characterized by relapsing-remitting inflammation of the gastrointestinal tract with a heterogeneous clinical phenotype.^1^^,^^2^ IBD profoundly impacts patients’ quality of life, resulting in disability and work productivity loss.^3^ Furthermore, IBD care puts considerable demands on healthcare resources and is associated with increasing medical costs.^4–6^

Close monitoring of disease activity, treatment response, and medication side effects are integral aspects of IBD management and are key to improving long-term outcomes.^7^ Traditionally, these evaluations necessitate frequent visits to healthcare facilities, causing inconvenience for patients, work productivity loss, and healthcare utilization. Recent advancements in eHealth technologies have shown to reduce the need for in-person hospital visits.^8^ Notable developments in eHealth are the integration of remote symptom monitoring and the incorporation of calprotectin home testing.^9^ While these innovations have the potential to transform IBD care, blood sampling remains a challenge. It is predominantly performed in the hospital or at off-site facilities, resulting in absence from work or other daily activities and travel expenses. Blood tests may detect disease activity and medication side effects and are therefore indispensable for disease monitoring in IBD.^7^ Important parameters include biochemical tests (C-reactive protein [CRP], liver enzymes, and creatinine) and hematology tests (hemoglobin, leukocytes, and thrombocytes). A home-based method for blood sampling that enables the assessment of all necessary blood parameters will fully implement remote monitoring of patients with IBD.

Capillary blood sampling via a finger prick at home offers a novel solution to bridge this gap in IBD remote care. Recent validation studies have demonstrated a high intermethod agreement between capillary blood sampling at home and traditional venous blood sampling via venipuncture in hospital.^10^^,^^11^ Furthermore, capillary blood sampling at home for therapeutic drug-level measurements in IBD patients resulted in high rates of successfully returned blood samples and high patient satisfaction.^12^ However, in these studies, capillary blood sampling was used to measure a small set of parameters, for which only small amounts of blood were needed. In addition, usability and patient satisfaction were assessed at merely one time point. Consequently, the methodology of these studies did not mimic standard care, for which repeated, elaborate blood tests over time are needed.

In this study, we evaluated the feasibility of capillary blood sampling at home and satisfaction with this method in IBD patients at several time points during regular care.

## Methods

### Study design and study population

In this prospective, single-center proof-of-concept study, 27 participants ≥18 years of age with a confirmed diagnosis of IBD (ulcerative colitis [UC], Crohn’s disease [CD], or IBD unclassified) and an indication for frequent blood tests (because of a switch in maintenance therapy) were consecutively recruited between October 2023 and March 2024 at the outpatient IBD clinic of the Gastroenterology Department at the University Medical Center Utrecht, The Netherlands. Patients who were physically, visually, or mentally impaired, unable to properly understand or read the Dutch language or unwilling to participate in this study were excluded. This study was approved by the local medical ethics committee and was conducted following the guidelines for Good Clinical Practice and the Declaration of Helsinki (2013, revised version). All patients provided verbal informed consent for their participation and informed consent was registered in the electronic patient file.

### Blood withdrawal and data collection

In this study, patients performed one supervised capillary blood sampling in the hospital and two self-employed capillary blood samplings at home at two time points. At baseline (time point 0 [T0], in hospital), patients visited the outpatient clinic, where they were trained by a skilled phlebotomist in accordance with the Clinical and Laboratory Standards Institute guideline GP42-A6: Procedures and Devices for the Collection of Diagnostic Capillary Blood Specimens (6th edition).^13^ Whenever the trainer and/or patient agreed that the competency requirements had been met, the patient could continue with the study. Initially, the puncture site was cleaned with a tissue with 70% isopropyl alcohol and afterward was left to air dry. The finger prick was then executed with the use of a BD Microtainer contact-activated lancet (blue, width and depth 1.5 mm × 2.0). A minimum of 500 μL capillary blood was first collected in a BD Microtainer MAP K2EDTA tube (0.5 mL), and a minimum of 375 μL capillary blood was then collected in a Greiner Minicollect Lithium Heparin (0.8 mL). Although one filled tube was also deemed sufficient, it requires manual redistribution of the blood by laboratory staff and is thus less efficient for routine implementation. Therefore, in case of insufficient blood flow, patients were first asked to puncture twice, and in the exceptional case of insufficient blood for the second tube, patients were still asked to hand in the EDTA tube sample. After collection, both tubes were capped and inverted 8 times.

Patients were then asked to fill in a short questionnaire, with questions regarding patient experience, satisfaction, and preference for blood sampling method (see text, Supplementary Material 1, which includes the questionnaire). Subsequently, patients received two self-sampling kits that each included all items and instruction materials necessary to perform two self-withdrawals with a finger prick at home. Instruction material included a letter with an elaborate, step-by-step description of the method, a checklist, and a QR code to a video in which the capillary blood sample method was demonstrated. See Supplementary Material 2 for a schematic overview of the study time points and procedures.

Baseline data on age, sex, height, weight, IBD disease phenotype (CD or UC), disease severity (Harvey-Bradshaw Index for CD, and Simple Clinical Colitis Activity Index for UC), and CRP and fecal calprotectin levels were collected. Baseline study characteristics of each patient (age, sex, diagnosis) and laboratory measurement results were retrieved from electronic patient files. Survey responses were collected using CASTOR EDC.^14^ At home, patients were asked to perform the capillary blood sampling at T1 (between two and six weeks from baseline) and T2 (between four and twelve weeks from T1), following standard care, and to send the blood samples directly by post to our hospital laboratory for analysis. After each self-withdrawal at home, patients were again asked to complete the short electronic questionnaire. A similar questionnaire was used for T0, T1, and T2.

### Transport and temperature data

We recently validated the stability of and comparison between capillary and venous blood samples for complete blood count (CBC)^14^ and routine chemistry analyses.^16^ Acceptable variation was demonstrated between venous and capillary measurements. Furthermore, acceptable stability was demonstrated for 48 hours between 4 °C and 30 °C for nearly all CBC parameters (lymphocyte and neutrophil counts excluded), and for all routine chemistry analyses.

To ensure that the temperatures to which blood samples were exposed during transport did not exceed the stability margins of 4 °C to 30 °C,^15^^,^^16^ each self-sampling kit contained a calibrated reusable temperature sensor provided by TubeSense. The temperature sensors were activated by the participant after blood withdrawal and transported back to the hospital along with the blood samples, and deactivated after arrival at the laboratory. For the self-obtained capillary blood samples at home, a medical envelope was provided, in which the blood samples were sent to the hospital via the Dutch postal service. When temperatures dropped below 4 °C, patients were asked to deliver the envelopes to a PostNL package point. Furthermore, patients were advised to avoid posting during the weekend. Medical envelopes were reusable and treated by the postal service as priority mail to reduce transit time.

### Sample preparation and analysis

Once the samples arrived at the hospital, the lithium heparin tubes were centrifuged for 5 min at 4000 *g* and plasma was transferred to analyzer cups (Tube-Top sample cups, Atellica Solution; Siemens Healthcare Diagnostics Inc.). Blood samples in EDTA tubes were presented directly to the hematology analyzer, which first automatically mixed them before pipetting samples. They were then measured for hemoglobin, hematocrit, leukocytes, and thrombocytes, and blood samples in lithium heparin tubes were measured for alkaline phosphatase, alanine aminotransferase, aspartate aminotransferase, creatinine, and CRP. In the exceptional case a patient only handed in an EDTA tube for analysis, the diagnostic laboratory was informed to manually perform both CBC as well as routine chemical analyses on the blood sample collected in the EDTA tube. Alkaline phosphatase could not be determined in such case. All assays, requested by the treating physician, were conducted at the Central Diagnostic Laboratory of the UMC Utrecht, which is accredited under ISO15189, using the Siemens Atellica CH Analyzer and the Abbott ALINITY hq hematology analyzer (Abbott Diagnostics), and were performed according to the manufacturer’s instructions. The maintenance of the analyzer adhered to the manufacturer’s recommendations, and the accuracy of results was assured through a strict quality control program that incorporated both internal and external quality control measures.

### Study outcomes

The primary study outcome was the proportion of successfully collected and returned capillary samples by patients at home, suitable for analysis in our hospital. A blood sample was determined successful when (1) the minimal required amount of blood was collected in either one or two tubes (500 μL for the EDTA tube, and 375 μL for the LH tube), (2) the time between sample withdrawal recorded by the participant and receipt in our hospital was less than 48 hours, and (3) the sample quality was sufficient, as assessed by the absence of blood clotting and a hemolysis index measured prior to analysis of ≤2.

Secondary outcomes were the patient’s experience concerning pain, satisfaction, time spent, and perceived practicality of the capillary blood sampling at home via the finger prick, as measured through the questionnaires (Supplementary Material 1). Pain perception during capillary blood sampling was assessed after blood collection using a numeric pain rating scale (0 indicating no pain, 10 representing the most severe pain).^17^ Perceived difficulty of the capillary blood sampling was assessed per step using a Likert-type scale,^18^ with a 5-point numeric rating scale, ranging from 1 for “very easy” to 5 for “very difficult.” Assessment of acceptability involved the use of the Net Promoter Score (NPS),^19^ which queries participants’ inclination to recommend a product or service to a friend or patient. This score employs an 11-point numeric rating scale, ranging from 0 for “not at all likely” to 10 for “extremely likely.” Responses falling between 0 and 6 are categorized as detractors, those at 7 and 8 as passives, and ratings of 9 and 10 as promoters. The NPS is then determined by subtracting the percentage of detractors from the percentage of promoters. Last, preference for blood sampling technique (venipuncture in hospital vs capillary sampling in a home setting) was assessed.

### Statistical analysis

Demographic and clinical characteristics of the study population were expressed as mean ± SD or median (interquartile range [IQR]), when appropriate, and proportions were presented as percentages. To assess the normality of the data, both visual methods, such as normal probability (Q-Q) plots and histograms, as well as statistical tests, specifically the Shapiro-Wilk test, were employed. Missing data was not imputed or corrected for, as this would affect the interpretation of our success rates. All statistical analyses were performed using IBM SPSS statistics version 29.0.1.

## Results

**Figure 1.**
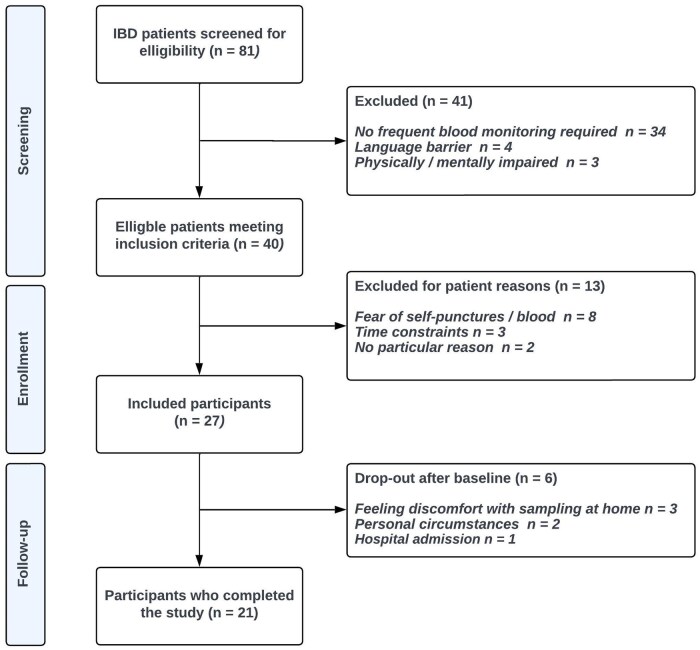
Inclusion and exclusion flowchart. IBD, inflammatory bowel disease.

A total of 27 patients were included in the study, with a participation rate of 68% (see Figure 1 for more details). Patients who refused to participate (n = 13) were 44.8 years of age (IQR, 25.5-62.0 years of age) and were predominantly of male sex (69% [n = 9]), and the most reported reason was a fear of self-puncture or blood (62% [n = 8]). The study population had a median age of 42.0 years (IQR, 30.0-54.0 years) and around half were female (52% [n = 14]). Most patients were diagnosed with CD (67% [n = 18]), of which 50% (n = 9) had a Harvey-Bradshaw Index score equal to or larger than 5. Baseline characteristics of the study population are shown in Table 1.

**Table 1.** Demographic and clinical characteristics of the study population (n = 27).

Characteristic	Data
**Age, y**	42.0 (30.0-54.0)
**Female**	14 (52)
**BMI, kg/m^2^**	26.3 ± 4.5
**IBD disease phenotype**	
** CD**	18 (67)
** UC**	8 (30)
** IBDU**	1 (4)
**Disease activity**	
** HBI**	
** <5, remission**	9 (50)
** 5-16, or mild disease**	9 (50)
**SCCAI**	163 (71-377)
**<5, inactive disease**	4 (50)
**>5, active disease**	4 (50)
**CRP, mg/L** ^a^	4.0 (0.5-18.0)
**Fecal calprotectin, µg/g** ^b^	163 (71-377)

Values are mean ± SD, n (%), or median [interquartile range].

Abbreviations: BMI, body mass index; CD, Crohn’s disease; CRP, C-reactive protein; HBI, Harvey-Bradshaw Index; IBD, inflammatory bowel disease; IBDU, inflammatory bowel disease unclassified; SCCAI, Simple Clinical Colitis Activity Index; UC, ulcerative colitis.

a CRP measured within 8 weeks before baseline.

b Fecal calprotectin measured within 8 weeks before baseline.

### Success rate and transport

**Figure 2.**
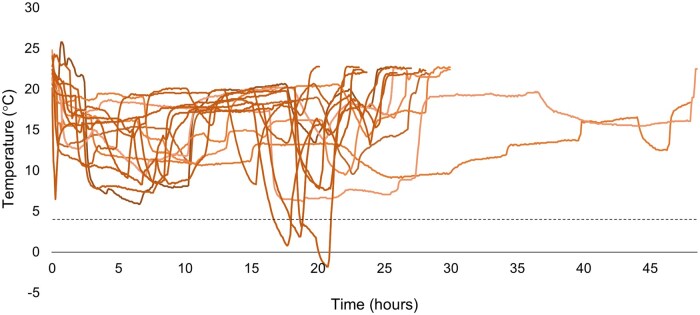
Temperature sensor data. Each line represents temperature data from one temperature sensor (n = 20), from the time of activation by the participant at home to the time of deactivation at the diagnostic laboratory. Thirteen temperature sensors were lost during the transport process.

A total of 69 blood samples were analyzed during the study, of which 27 were obtained in hospital at T0 and 21 at T1 and T2 each. Median follow-up time was four weeks at T1 and eight weeks at T2. Six (22%) patients ended their participation prematurely, for various reasons (Figure 1). Success percentages, percentage of participants that were able to fill both the EDTA tube and the lithium heparin tube with capillary blood, and median transport times between withdrawal at home and arrival at the laboratory are shown in Table 2. Of the four unsuccessful blood samples at T1, one took more than 48 hours to be transported to the hospital, one did not contain the minimally required amount of blood for analysis, one contained a blood sample of insufficient quality (blood clot found in sample), and one contained a blood sample of both insufficient quantity and quality. At T2, one sample was of insufficient quality. Furthermore, temperature data from 29 temperature sensors were collected. The remaining 13 sensors did not collect data due to malfunction or inappropriate activation, or because they were lost during the sending process or at the laboratory. Three samples were exposed to temperatures exceeding the lower stability limit of 4 °C (Figure 2), of which one sample was shortly exposed to temperatures below 0 °C. The mean hemolysis index at T0 was 0.90 ± 0.32, at T1 was 1.30 ± 0.68, and at T2 was 1.20 ± 0.42.

**Table 2.** Capillary blood sampling success rates and transport time.

	T1	T2	Total T1 and T2
**Success percentage**	17/21 (81)	20/21 (95)	37/42 (88)
**Both EDTA and lithium heparin tube filled**	15/21 (71)	19/21 (91)	34/42 (81)
**Transport time, h**	29 (24.0-37.5)	26.3 (23.0-29.3)	26.4 (23.8-30.3)

Values are n/n (%), or median (interquartile range).

Abbreviation: T, time point.

### Patient satisfaction and experiences

The questionnaire at both time points had a response rate of 100% (n = 21 of 21). The live training in the hospital was perceived as “very clear” by 92% of the patients, the instruction letter was perceived as “very clear” by 81% of the patients, and the instruction video was perceived as “very clear” by 71% of those who watched it. At T1, 61% (n = 13 of 21) of the patients preferred capillary sampling at home over venous sampling at the hospital. At T2, the proportion of patients who preferred capillary sampling at home slightly decreased to 57% (n = 12 of 21). At both T1 and T2, the majority of patients expressed normal to high satisfaction with the capillary sampling method at home (71% at T1 [n = 15 of 21] and 67% at T2 [n = 14 of 21]). Preference for capillary sampling at home varied with age: at T1, 60% (n = 3 of 5) of 20- to 29- year-olds, 86% (n = 6 of 7) of 30- to 43- year-olds, and 44% (n = 4 of 9) of 44- to 75- year-olds preferred capillary sampling at home, and at T2, 80% (n = 4 of 5) of 20- to 29- year-olds, 71% (n = 5 of 7) of 30- to 43- year-olds, and 33% (n = 3 of 9) of the 43- to 75-year-olds preferred capillary sampling at home over venous sampling at the hospital. A similar trend was seen for patient satisfaction at T2: 80% (n = 4 of 5) of 20- to 29-year-olds, 71% (n = 5 of 7) of 30- to 43-year-olds, and only 44% (n = 4 of 9) of 44- to 75-year-olds expressed normal to high satisfaction with the novel method. Last, the NPS score was positive and very similar at both time points (+23.9 in T1 and +23.8 in T2), meaning the majority of patients would recommend the capillary finger prick at home to other patients.

**Figure 3.**
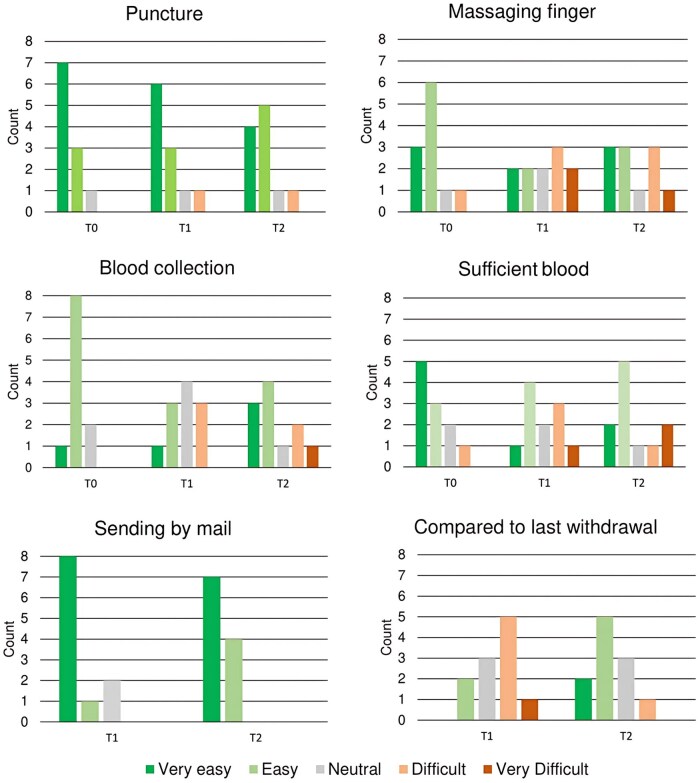
Bar diagram detailing questionnaire items related to patient satisfaction and tolerability regarding capillary blood withdrawal. The bars represent the total count per answer category reported by patients at time point 0 [T0], T1, and T2.

The capillary finger-prick method at home was not considered painful, with similar numeric pain rating scale scores of 1.0 (IQR, 0.0-3.0) at T1 and 1.0 (IQR, 0.0-3.0) at T2. At home, the majority of patients were assisted by their peers to perform the capillary withdrawal at both time points (71% at T1 [n = 15 of 21] and 57% at T2 [n = 12 of 21]), and a small percentage of patients punctured twice (38% at T1 [n = 8 of 21] and 19% at T2 [n = 4 of 21]). Last, patients rated various aspects and steps of their experience with the capillary blood withdrawal on a 5-point Likert-type scale at the different time points (Figure 3). The majority of patients reported puncturing with the device and returning the package by mail to the hospital as “easy” or “very easy.” Most difficulty was experienced with massaging the finger, collecting the blood in the tube, and assessing whether sufficient blood was collected. At T2, 15 (71%) of 21 patients rated the difficulty of the entire capillary withdrawal performance as easier compared with the previous withdrawal at T1. Answers to open questions are shown in the table in Supplementary Data Content 3.

## Discussion

In this proof of concept study, we demonstrated the feasibility of finger prick–based capillary blood sampling at home for monitoring IBD patients, with high success percentages. To our knowledge, this is the first study assessing capillary sampling at home for both CBC and routine chemistry measurements and at more than 1 time point. As a result, this study represents the actual clinical setting where capillary sampling might be implemented in the future. Furthermore, this is the first study with temperature measurements during transport, giving insight into the conditions to which blood samples are exposed during transport.

We reported success percentages of 81% at T1 and 95% at T2, with an overall success percentage of 88%. This is slightly lower compared with similar studies, with success percentages of 92% to 98%. However, the amount of blood collected per sample in these studies was approximately tenfold lower.^10–12^ The success rate at T2 was higher compared with T1. This possibly represents a learning effect, that previous studies have not yet explored. A learning effect is further supported by the survey results pointing out that most patients experienced the second withdrawal as easier than the first withdrawal, and that fewer patients at the second withdrawal needed to puncture twice, needed assistance from their peers, or managed to fill only one tube. Improvement over time is a strong argument in favor of the implementation of capillary sampling at home, especially for patients who require frequent blood tests for years due to their chronic illness, such as IBD patients. Furthermore, in clinical scenarios in which only one set of parameters is sufficient, or in which laboratory capacity allows for manual handling, a single tube can still provide clinically meaningful results. Filling a single tube with blood is easier to perform, as the tube can then immediately be inverted, which lowers the risk of blood clots. Therefore, in a setting in which only one tube is necessary, the observed success rate and perceived feasibility could thus be even higher.

Slightly more than half of patients expressed normal to high satisfaction with the novel method and expressed preference for the novel method over venous blood sampling at the hospital. Furthermore, our NPS of approximately +24 at both time points is reasonably positive but lower than in some previous reports. The reason for this smaller proportion compared with similar studies^10^^,^^12^^,^^20^^,^^21^ might be the more rigorous protocol with a much larger volume of blood that needed to be collected in not one but two tubes, to monitor both CBC and routine chemistry parameters. A higher capillary blood yield requires more time and a more advanced withdrawal technique and is thereby more prone to mistakes. However, this also provides a more realistic indication of patient acceptance for intensive monitoring than investigations with smaller specimen volumes. For the collection of blood in two tubes, an extra pair of hands from a peer can make the withdrawal much easier. This is further supported by the high rate of peer assistance (71% at T1, 57% at T2), which suggests that this method may not be appropriate for individuals living alone or with limited social support. This dependency might undermine a key advantage of home collection, namely greater convenience and autonomy from healthcare centers. However, optimization of the withdrawal technique and device might resolve this problem and increase patient autonomy in the near future. When considering different age groups, the proportion of younger patients who preferred the novel method seemed larger than that of older patient groups. Because younger patients generally adopt busier lifestyles (eg, working and raising children), they possibly have more to gain from a method enabling blood sampling at home. Moreover, because capillary blood sampling requires sufficient fine motor skills and adequate peripheral blood flow, both influenced by age, older patients may experience more difficulty in capillary sampling. This finding may indicate the continued need for venous sampling in older IBD patients.

Most difficulty was experienced with the blood collection technique, especially massaging the finger and ensuring enough blood was collected, similar to the observations in the study by Otten et al.^12^ Several promising capillary blood collection devices have been developed to overcome these issues, which are less invasive and enhance blood release and consistent blood volume. The major disadvantage of these devices is that they are often expensive and that the total capillary blood yield is generally lower.^22^ Most patients did not experience difficulty or pain when performing the finger prick with a lancet. It is important to recognize that most of these patients had previous experience with self-injections for administering medication. Furthermore, sending the blood samples to the hospital by mail was considered easy by the majority of patients. The average transport time was between 24 and 48 hours, which lies within the stability margins of both CBC^15^ and routine chemistry^16^ parameters. One sample, shipped during the busy Christmas holiday season, had a transit time of >48 hours, yet still retained valid outcomes. This finding suggests that there is potential to extend transport times in future implementations. Furthermore, the majority of samples were exposed to temperatures within the stability margins. Thirteen temperature sensors did not collect any data due to malfunction or user activation issues or because of being lost during the process. Of those that did collect data, three exceeded the lower temperature margins, which highlights the necessity for better shipping protocols. However, they were exposed to the cold (<4 °C) for only one to three hours, and the effect on the stability of the blood parameters was most likely negligible. One sample was shortly exposed to temperatures below 0 °C, but this did not result in excessive hemolysis. The study period took place during the winter season in the Netherlands; therefore, exposure to low temperatures might have occurred during transport by the postal service, or when patients did not deliver the envelopes to a package point as instructed. To address the issues with temperature sensors, a newer generation of sensors, now commercially available, could be used for future implementation. These no longer require manual activation and can be directly attached to the return envelope, thereby improving reliability and reducing user errors. Last, our findings demonstrate increased hemolysis in samples derived from at-home sampling. Even though this is expected due to sampling technique variation and increased transport time, it is important to consider the potential impact on individual assays. Hemolysis may affect parameters such as potassium, lactate dehydrogenase, and aspartate aminotransferase, with clinically relevant interference generally occurring at a hemolysis index ≥3 to 4. Previous work^16^ showed that finger-prick sampling is associated with a modest rise in hemolysis index, without compromising result reliability. Laboratories accredited under ISO15189:2023, such as the laboratory in this study, are required to define assay-specific acceptance and exclusion criteria, ensuring that unreliable results are either withheld or reported with appropriate comments. Therefore, the increased hemolysis observed in our study samples is unlikely to have affected the validity of the reported results.

Further innovation and implementation studies are needed with an optimized sampling device but for an acceptable price. This will enable future implementation of capillary sampling at home for routine blood tests in IBD patients on a large scale. Combined with remote fecal calprotectin testing and mobile applications that track patient-recorded outcome measures, implementation would allow IBD patients to manage their disease almost entirely in a home setting, which saves them time and money. It is conceivable that this will reduce disease burden and positively impact quality of life, especially in the younger IBD patient population. In addition, the implementation of capillary sampling at home would be in line with current trends and policies aimed at reducing healthcare consumption and costs through remote care.^23^

A few limitations of this study are important to recognize. First, the study’s generalizability is limited by a modest sample size due to a participation rate of 68% and a dropout rate of 22%. Of the eligible patients that declined participation, 62% cited fear of self-puncture or blood as the main reason. This introduces a potential selection bias toward highly motivated and confident users, which could lead to an overestimation of the real-world uptake and success of at-home blood sampling. Broader implementation might face substantial adoption barriers that need to be carefully considered in participant selection and support strategies. However, by describing the reasons for dropping out or declining in a transparent and detailed manner, this study provides useful information for future implementation (eg, when deciding which patients are more inclined to adopt this novel method). Future implementation efforts should consider tailored support strategies, such as psychological support or alternative sampling options, to address self-sampling anxiety. It is also relevant to note that patients treated in our hospital were allowed to have blood drawn at a local laboratory close to their home, which may have reduced the incentive to participate. In many other centers, this option is not available, and in such circumstances participation rates may therefore be higher. Furthermore, even though the amount of capillary blood collected from patients in this study was larger compared with similar studies, it was specifically tailored to the parameters necessary for routine IBD disease monitoring. In several patients, a limited amount of blood was collected due to insufficient flow or other technical difficulties. The amount of patients collecting a sufficient amount of blood and reporting the blood collection as difficult did not decrease at T1 and T2, highlighting that these steps during self-sampling remain most challenging. Optimized withdrawal techniques are needed to ensure that blood collection is less difficult to perform, thereby improving patient comfort and allowing the analysis of a larger set of parameters in the near future. One example of such an optimization would be to create a larger, funnel-like opening at the top of the blood tube, enhancing the visibility of the volume indicator, or opting for a device that can apply a vacuum over the lanced site to enhance blood flow.^22^ Furthermore, this study explored capillary sampling with one validated device, which makes it unclear whether challenges stem from the apparatus or from home collection. This highlights the need for a future study in which different validated devices are compared in parallel or sequentially during a longer time frame. Last, due to the feasibility-focused design, an economic evaluation was beyond the scope of this study and a formal cost-effectiveness analysis was not conducted. However, we recognize that comprehensive cost-effectiveness analyses, accounting for direct healthcare and device costs, as well as laboratory workflow expenses in relation to reductions in laboratory visits and patient-incurred costs (eg, travel time, loss of work productivity), will be critical in future studies to support large-scale implementation and inform healthcare policy decisions.

To conclude, capillary sampling at home is a promising method for monitoring IBD patients. This study was the first to simulate clinical practice, including both CBC and routine chemistry parameters at different time points at home. In order to achieve successful deployment and broad application in the future, attention needs to be paid to individual preferences, patient characteristics, and intensive support structures. This study shows a learning effect of home sampling, supporting the value of continued practice and training. At the same time, recurring challenges with specific tasks—especially finger massage and specimen volume measurement—show that improvements must also address technical aspects of the apparatus, not just user experience. Device and technique optimization are needed to facilitate higher patient satisfaction and to ensure a failproof capillary sampling method. With these optimizations, we believe that the combination of at-home blood sampling with telemedicine and calprotectin home testing can bring us one step closer to fully implement remote monitoring of IBD patients.

## Supplementary Material

izaf240_Supplementary_Data

## Data Availability

The data underlying this article will be shared on reasonable request to the corresponding author.
