# Supplementary material for: Implementation of a Capillary Blood Self-Sampling Technique at Home for Monitoring of Patients With IBD
Source: Inflamm Bowel Dis. 2025 Oct 31;32(2):282–9. doi: 10.1093/ibd/izaf240 (PMC12857424; doi:10.1093/ibd/izaf240)
Supplement: izaf240_Supplementary_Data [file izaf240_supplementary_data.zip › Supplementary Material 3_revised_clean.docx]

**Supplementary Material 3: Table with answers to open question**

Question: “*Were there any specific difficulties that you ran into when performing the self-sampling?”*

| **Answers** |
| --- |
| *“Light-headedness.”* |
| *“After swirling the first tube, it becomes difficult to fill the second one. In my case, this didn’t work, and a second puncture was necessary. This is not desirable.”* |
| *“It was difficult to find a good position for my hand. My nails were not clipped, and blood got behind them.”* |
| *“I found it difficult to see if the blood was properly entering the tube due to the position of my hand.”* |
| *“Dizziness, and I couldn’t perform the puncture in the right manner.”* |
| *“The blood didn't flow easily.”* |
| *“There was a possibility that it was colder than 5 degrees outside and that I would have to go to a postal point to drop off the tubes - which I didn't have time for that day.”* |
| *“The first tube went reasonably well. For the second one, I asked for help from a neighbor (a nurse) and it went better.”* |
| *“Occasionally, it didn't go so well, but I'm also an impatient person.”* |
| *“The tube is very narrow and I spilled a few drops, making the sides of the tube and the sticker dirty. Also, it was colder than 5 degrees outside, so I decided to put it in the hospital mailbox just to be safe, so I still made the trip to the hospital. Luckily I had to go to the hospital anyway to pick up medication from the locker, so it wasn’t extra effort. Lastly, it was a bit annoying during sports afterward because my finger had become sore (I do rock climbing).”* |
| *“Still, I felt a mental barrier to puncture myself. And the blood just didn't want to flow.”* |
| *“Filling the second tube without an additional puncture was not possible in my case. I found a second puncture undesirable and therefore didn’t perform it.”* |
| *“It wasn't possible to fill the tubes quickly; it was very difficult. I have now asked the neighbor (a nurse) for help again, just like last time”* |
| *“The opening of the tube is really too narrow, causing many drops to fall outside rather than into the tube.”* |
| *“I tried, but more blood ended up outside the tube than inside. So it didn't work, and now I still have pain in my finger all day long – like a papercut. That's annoying when working on the computer. This problem doesn’t occur with a puncture in the arm.”* |
| *“Through my own fault, I pulled the label off the thermometer too early. That was very inconvenient. In addition, it takes some effort to find someone able to help with the blood withdrawal before 5 p.m. on a working day, if you both have a job and leave home early (between 6:00 AM and 6:30 AM). Doing it at work with a colleague felt a bit strange.... So I ended up doing the blood test at 6:30 AM on an empty stomach (which was not a wise decision).* |
| *“The blood didn't drain easily”* |
| *“My finger remained painful for a long time after injection”* |
| *“Even though I warmed my hand under the tap, the drops came slowly. After filling the first tube, I had to puncture a second time, which was less successful. So I wasn’t able to fill two full tubes this time.”* |
| *“I spent a little too much time cleaning and applying a bandaid, and started turning the green tube a little too late. The blood may have started to clot slightly.”* |
| *“It was not possible to fill the tubes quickly, it was very difficult. I have now asked the neighbor (nurse) for help again, just like last time.”* |
| *“It's nice to have control over the timing of the blood sampling. This time the blood came more slowly and both my hands were covered in blood because it stuck to the top of the tube and didn't fall in... that was a bit awkward. Also, with a full-time job, finding the right moment remains difficult—same for my partner. We both stayed home so we could mail it the same day, which somewhat reduces the benefit and time savings.”* |
| *“The opening of the tube is really too narrow. Many drops didn’t fall into the tube but hit the outside. As a result, the tube got heavily contaminated, and there weren’t enough drops to properly fill the second tube. The discomfort also lasted longer. Filling the second tube takes time, so the first one might start to clot. In any case, a wider opening would be appreciated.”* |
