# Supplementary material for: Implementation of a Capillary Blood Self-Sampling Technique at Home for Monitoring of Patients With IBD
Source: Inflamm Bowel Dis. 2025 Oct 31;32(2):282–9. doi: 10.1093/ibd/izaf240 (PMC12857424; doi:10.1093/ibd/izaf240)
Supplement: izaf240_Supplementary_Data [file izaf240_supplementary_data.zip › Supplementary material 1 .docx]

**Supplementary Material 1: timepoint 1 questionnaire**

*Questionnaires at T0 and T2 were the same apart from questions 1 and 2.*

**Date and time bloodsample:** date ../../…., time: ..:.

1. **How clear was the instruction letter that you received with the self-sampling kit?**

- Very clear
- Clear
- Not clear or unclear
- Unclear
- Very unclear
- I did not read the instruction letter

1. **How clear was the instruction video on YouTube (please refer to the URL and QR code in the instruction letter)?**

- Very clear
- Clear
- Not clear or unclear
- Unclear
- Very unclear
- I did not watch the video

1. **How much pain did you experience during the self-sampling?**
   **(Please choose the number that fits the best)**

- 0 – No pain
- 1
- 2
- 3
- 4
- 5
- 6
- 7
- 8
- 9
- 10 – Extreme pain

1. **How did you experience the pain / discomfort of the self-sampling compared to the venous sampling from your arm at the hospital?**

- Less painful / less uncomfortable
- Equally painful / equally uncomfortable
- More painful / more uncomfortable

1. **How satisfied are you with the self-sampling method at home?**

- Very satisfied
- Satisfied
- Neutral
- Dissatisfied
- Very dissatisfied

1. **How easy or difficult was it to puncture the finger with the lancet?**

- Very easy
- Easy
- Neutral
- Difficult
- Very difficult

1. **How easy or difficult was it to massage the blood from your finger?**

- Very easy
- Easy
- Neutral
- Difficult
- Very difficult

1. **How easy or difficult was it to collect the blood droplets in the tube?**

- Very easy
- Easy
- Neutral
- Difficult
- Very difficult

1. **How easy or difficult was it to fill the tubes with enough blood?**

- Very easy
- Easy
- Neutral
- Difficult
- Very difficult

1. **How easy or difficult was it to return the blood to the hospital by post?**

- Very easy
- Easy
- Neutral
- Difficult
- Very difficult

1. **How easy or difficult was the blood sampling compared to the previous self-sample?**

- Very easy
- Easy
- Neutral
- Difficult
- Very difficult

1. **Were there any specific difficulties that you ran into when performing the self-sampling? You can elaborate further below.**

……………………………………………………………………………………………………………………………………..

……………………………………………………………………………………………………………………………………..

1. **Did anyone (e.g. your partner, roommate, or family member) help you during the self-sampling?**

- Yes
- No

1. **How many times did you have to puncture your finger?**

- Once
- Twice

1. **If you had a choice, which method for blood sampling would you prefer?**

- In the hospital via the standard method (sample from the arm)
- At home via a finger prick (self-sampling)
- At the hospital via a finger prick
- No preference

1. **How likely is it that you would recommend this self-sampling method at home to other patients?**

- 0 = Very unlikely
- 1
- 2
- 3
- 4
- 5
- 6
- 7
- 8
- 9
- 10 = Very likely
