# Supplementary figures and images for: Implementation of a Capillary Blood Self-Sampling Technique at Home for Monitoring of Patients With IBD
Source: Inflamm Bowel Dis. 2025 Oct 31;32(2):282–9. doi: 10.1093/ibd/izaf240 (PMC12857424; doi:10.1093/ibd/izaf240)

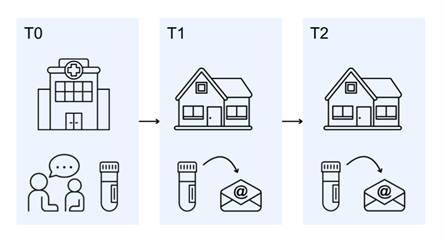

Supplement: izaf240_Supplementary_Data [file izaf240_supplementary_data.zip › Supplementary material 2.tif]
